# Injectable platelet rich fibrin versus low-level laser therapy for orthodontic tooth movement: A clinical split-mouth study

**DOI:** 10.6026/973206300213345

**Published:** 2025-09-30

**Authors:** Sunilkumar Nagmode, Pranoti Gade, Kunal Patil, Hrushikesh Aphale, Payal Barse, Ekata Jaindesarda

**Affiliations:** 1 Department of Orthodontics and Dentofacial Orthopaedics, SMBT Dental College and Hospital Ghulewadi, Sangamner, Maharashtra, India; 2 Department of Periodontology, SMBT Dental College and Hospital Ghulewadi, Sangamner, Maharashtra, India

**Keywords:** Platelet rich fibrin, low-level laser therapy, orthodontic tooth movement, split mouth study

## Abstract

Accelerating tooth movement during orthodontic treatment is a growing focus in clinical orthodontics. Techniques like injectable platelet rich fibrin (i-PRF) and Low-Level Laser Therapy (LLLT) have demonstrated encouraging results. Hence, 20 patients aged 18-25 years were enrolled and one side of their maxillary arch was injected with intraligamental and submucosal i-PRF and the opposite side with LLLT (980nm, 100mW). Measurements were taken using digital vernier calliper on distal surface of canine to mesial surface of second premolar monthly for four months. The mean rate of space closure on i-PRF side (right) was significantly higher (2.85 ± 0.47 mm) than LLLT side (2.05 ± 0.28 mm; p<0.001). The left side (control group) showed no significant difference (p=0.833). Thus, i-PRF is more effective than LLLT in accelerating orthodontic tooth movement. Further multicentre trials are recommended.

## Background:

Orthodontic tooth movement (OTM) is a dynamic process driven by biological response to external forces that disrupt the physiological equilibrium within dentofacial complex [1]. To expedite this natural process, various methods have been explored, with recent emphasis on two promising approaches: local injection of platelet rich fibrin (i-PRF) and low-level laser therapy (LLLT). Platelet rich fibrin (i-PRF) is characterised by small volume of plasma with an autologous concentrate of platelets. This technique is regarded as one of the most recent and effective ways to accelerate orthodontic tooth movement [2]. Both osteoblastic and osteoclastic activities are significantly stimulated by the abundance of growth factors in i-PRF [3]. Furthermore, differentiation, activation, and survival of different bone cells are facilitated by its abundant cytokine content [4]. These aspects together advocate that i-PRF can influence and enhance the biological mechanism involved in OTM. LLLT has been described using terms such as "soft laser therapy," "cold laser," "low energy laser therapy (LELT)," and "low intensity laser therapy (LILT)," emphasizing its gentle application [5]. LLLT provides a physical method to accelerate orthodontic tooth movement through its biostimulatory effects [6]. This process entails encouraging bone remodelling by enhancing proliferation of osteoblasts and osteoclasts on tension and pressure side respectively. Furthermore, LLLT has demonstrated the ability to promote collagen synthesis, which further adds to the overall efficacy of this method [7]. Application of i-PRF and LLLT are acknowledged as innovative and efficient methods of increasing tooth movement during orthodontic treatment. These techniques utilize the biological reactions present in the dentofacial complex, presenting exciting possibilities for advancements in orthodontic treatment methods. While extensive research has been conducted on both approaches, there is a lack of direct comparative studies. Therefore, it is in interest to study and compare the effectiveness of i-PRF and LLLT in tooth movement acceleration in orthodontic treatment using a split-mouth clinical model.

## Materials and Methods:

A prospective split-mouth study was conducted at the Department of Orthodontics and Dentofacial Orthopedics. Ethical approval was obtained from institutional review board (Ethics No: EC/NEW/INST/2021/1810) and informed consent was taken by all the participants. 20 participants fulfilled the criteria and were assigned randomly to two groups. Computer-generated random list was created and allocation concealment was done with opaque envelopes. Group 1 was injected with i-PRF on the right side whereas, Group 2 received low level laser therapy on the right side. The left sides (contralateral side) served as control. Patient aged 18-25 years with permanent dentition who were indicated for bilateral premolar extraction were included in this study. Participants who were undergoing orthodontic treatment for the first time and maintained good oral hygiene to receive MBT 0.022" slot with mini-screws were considered. We excluded anyone with systemic disorders or syndromes, tooth anomalies or long-term use of any medication. Complete preoperative records were acquired and all participants received traditional orthodontic treatment that included completing first phase of the treatment i.e.; extraction of first premolars followed by aligning and levelling with MBT 0.022 slot. En-masse retraction was started after alignment with the aid of E-chain and mini-screws. E chain was tied around the canine to TAD (Temporary Anchorage Device) that was inserted between second premolar and first molar.

## Application of i-PRF:

i-PRF was freshly prepared for the procedure using single spin centrifugation method (Figure 1 - see PDF). Before application of i-PRF, local anaesthesia was applied for pain control. The distobuccal, middle, and distopalatal areas of the distal surface of canine were injected with PRF intraligamentally together with buccal and palatal submucosal injections on the other side of the arch (maxillary) for the same patient. The i- PRF was injected at interval of 0, 21, and 42 days. Impression was made before starting the retraction phase, and at the end of every month for 4 months. A digital vernier calliper was used to measure the distance between mesial surface of second premolar and canine's distal surface on a dental cast. Measurements were recorded on both the sides of the arch.

## Application of LLLT:

One side of the arch (maxillary) received weekly low level laser therapy for the first month, then every other week for the remaining four months, operated at a wavelength of 980nm. 100 mW of power output and 1 J/cm^2^ of energy density in continuous mode were used. The laser's flexible fiber optic cable was attached to handpiece and the tip was held perpendicular in direct contact with mucosa. LLLT was applied at three locations on the canine's buccal and palatal surfaces, with a 10-second irradiation time at the cervical, middle, and apical levels. A digital vernier calliper was used to analyse study models to measure the distance from distal surface of canine to mesial of second premolar (Figure 2 - see PDF). The measurements were recorded on both sides of the arch.

## Statistical analysis:

Statistical analysis was performed with SPSS software version 21. P value of less than 0.005 was considered significant. Considering the split-mouth study design, comparisons between Group A (i-PRF) and Group B (LLLT) at specific time intervals was conducted with unpaired t-test.

## Results:

20 participants with age between 18-25 years were included in this study with 10 individuals divided into two groups randomly. A split mouth study was designed, where we randomly allocated i-PRF to group 1 and LLLT to group 2 on the right side. The left sides acted as controls for both the groups. The i-PRF (A group) showed significantly higher rate of space closure (2.85 ± 0.47 mm) on the right side compared to LLLT (B group) (2.05 ± 0.28 mm) (p < 0.001) as shown in Table 1 (see PDF). No significant difference was observed between i-PRF (2.15 ± 0.52 mm) and LTTT (2.1 ± 0.51 mm) groups on the left sides (p= 0.833). As depicted in Figure 3 (see PDF), in intra group comparison i-PRF showed significantly greater space closure rate on right side than left side (p=0.006). No such difference was found with LLLT group.

## Discussion:

The present split-mouth study aimed to compare rate of orthodontic tooth movement (OTM) using injectable PRF and LTTT. Our study revealed that i-PRF considerably accelerated orthodontic tooth movement on the right side compared to LLLT which showed no visible difference in orthodontic tooth movement on the right side.

## Orthodontic tooth movement with i-PRF:

The space closure rate from T0 to T1 with i-PRF showed higher closure rates on the right side. This is attributed to the mechanism by which i-PRF promotes bone remodelling and osteoclastic activity via growth factors like PDGF and TGF-β [8]. Güleç *et al.* demonstrated that platelet-rich plasma accelerated tooth movement in rat models through histomorphometric changes [9]. A randomised control trial by Priya *et al.* documented significantly enhanced space closure rate in orthodontic treatment with injections of i-PRF [10]. Similarly, a meta-analysis by Singh *et al.* [11] validated that PRF-based injections consistently accelerates orthodontic tooth movement rates. Likewise, our results align with these studies that i-PRF accelerated orthodontic tooth movement. The study by Talar *et al.* found that while platelet-rich fibrin (i-PRF) injections didn't significantly accelerate tooth movement, there was a temporary increase at the second month [12]. This implied that PRF's impact on tooth movement might not last long and that continuous acceleration of retraction would require repeated injections. In agreement with our findings, a recent clinical study by Nasef, AE *et al.* [13] also highlighted that i-PRF injections significantly enhanced the rate of orthodontic space closure. This effect was attributed by the authors to a sustained release of growth factors and improved cellular response at the tension side, providing further biological support for the use of PRF in orthodontic treatment.

## Orthodontic tooth movement with LLLT:

Low level laser therapy (LLLT) accelerates orthodontic tooth movement by enhancing levels of RANKL in PDL leading to increased osteoclastogenesis and increased M-CSF levels [14]. Although LLLT has shown to stimulate bone remodelling, clinical results vary in this context. A meta-analysis and systematic review revealed LLLT's limited impact [15]. However, our study did not find statistical inter-group differences with LLLT. This may be a result of variation in parameters like wavelength, frequency, and dosage. Compared to higher densities, lower energy densities of laser (5 and 8 J/cm) was seemingly more effective. Thus, the negative result is likely a consequence of suboptimal parameter settings, rather than the method's inherent limitations. Al-Fakhry *et al.* [8] demonstrated enhanced alveolar bone density, decreased relapse, and accelerated orthodontic tooth movement. Thus, increased stability after orthodontic tooth movement after i-PRF gives long term benefits. Routine orthodontic procedures combined with injectable platelet rich fibrin (i-PRF), a patient-derived, low cost, and minimal invasive procedure can hasten the healing process. Also, i-PRF is beneficial because it reduces the duration needed for treatment procedures. Being effective, this study has certain limitations like, smaller sample size, laser parameter optimization also only one i-PRF application. To further validate the results, further research should focus on increased sample size, standardization, and optimization of LLLT parameters.

## Conclusion:

We show that i-PRF and LLLT offer biologically sound options to accelerate orthodontic treatment, although i-PRF notably enhanced canine retraction compared to LLLT. Since it is minimally invasive and uses the patient's own blood, i-PRF may be the better option. LLLT, though biologically plausible, showed no significant clinical effect under current protocol parameters. Clinicians should weigh the benefits of both the procedures for efficient treatment.
